# The Therapeutic Efficacy and Molecular Mechanisms of *Artemisia argyi* Essential Oil in Treating Feline Herpesvirus Infection via Nasal Drops

**DOI:** 10.3390/vetsci12020080

**Published:** 2025-01-23

**Authors:** Tian Wan, Jinze Li, Jiayi Liu, Yingxin Chen, Yihao Guo, Xianjie Deng, Xingyao Li, Jiachen Bi, Chongchong Hu, Jianyu Chang, Kai Fan

**Affiliations:** College of Veterinary Medicine, China Agricultural University, Beijing 100193, China; wantian@cau.edu.cn (T.W.); s20213050846@cau.edu.cn (J.L.); jiayiliu_7@cau.edu.cn (J.L.); irene1020747@163.com (Y.C.); yhguo0101@163.com (Y.G.); jerryteng19990510@gmail.com (X.D.); 732392798@163.com (X.L.); bjc990826@163.com (J.B.); kiligela@163.com (C.H.); changjianyu@cau.edu.cn (J.C.)

**Keywords:** *Artemisia argyi* essential oil, feline herpesvirus, feline viral rhinotracheitis, IL-17 signaling pathway, immune modulation, inflammation control, tissue protection

## Abstract

This study aimed to explore the therapeutic potential and mechanisms of Artemisia argyi essential oil (AAEO) in managing feline herpesvirus type 1 (FeHV-1) infections. FeHV-1, the causative agent of feline viral rhinotracheitis (FVR), leads to severe respiratory and systemic complications in cats. In this study, 35 cats were divided into blank, FeHV-1 infection, and AAEO treatment groups (high, medium, and low doses). In vivo experiments demonstrated that AAEO alleviated clinical symptoms, reduced tissue damage, and modulated immune responses. The AAEO-treated groups showed higher survival rates, stabilized body temperatures, and less severe weight loss compared to the FeHV-1 group. Histopathological analysis revealed improved integrity in nasal, tracheal, and bronchial tissues. Transcriptomic and proteomic analyses identified critical pathways, such as IL-17 signaling, influenced by AAEO treatment, highlighting its role in suppressing inflammation and protecting tissue integrity. In vitro assays revealed that AAEO has concentration-dependent cytotoxicity in feline kidney cells (F81) and provides protective effects when used as a pre-treatment. These findings suggest that AAEO enhances host immune defenses and mitigates FeHV-1-induced damage through immune modulation and tissue protection.

## Simple Summary

This study explored the therapeutic potential of Artemisia argyi essential oil (AAEO) in managing feline herpesvirus type 1 (FeHV-1) infections. FeHV-1, the causative agent of feline viral rhinotracheitis (FVR), leads to severe respiratory and systemic complications in cats. In this study, cats treated with AAEO showed reduced clinical symptoms, improved tissue integrity, and better immune responses. AAEO-treated groups had higher survival rates, stabilized body temperatures, and less weight loss compared to untreated FeHV-1-infected cats. Tissue analysis revealed better integrity in nasal, tracheal, and bronchial tissues. AAEO was also shown to suppress inflammation and protect tissues by modulating immune pathways such as IL-17 signaling. These results suggest that AAEO enhances immune defenses and reduces FeHV-1-induced damage through immune modulation and tissue protection.

## 1. Introduction

*Feline herpesvirus type 1* (FeHV-1), also referred to as *Feline alphaherpesvirus 1*, is a virus from the genus *Alphaherpesvirus* within the subfamily *Alphaherpesvirinae*. Currently, only one serotype has been identified [1]. FeHV-1 shares many biological characteristics with other alphaherpesviruses, such as herpes simplex virus (HSV), varicella zoster virus (VZV), and canine herpesvirus-1 (CHV-1), including the ability to infect skin, mucous membranes, and nervous tissues and to remain latent within the host [2].

FeHV-1 is one of the main pathogens causing feline viral rhinotracheitis (FVR), which is widely spread worldwide and poses a serious threat to feline health. FeHV-1 is a virus that specifically targets epithelial-like cells, inducing cytopathic effects (CPEs). Studies have shown that FeHV-1 infection induces apoptosis in a dose- and time-dependent manner, primarily through the intrinsic pathway involving mitochondrial damage [3]. The tissue damage caused by FeHV-1 is primarily concentrated in the nasal septum, turbinates, nasopharynx, tonsils, and upper respiratory tract mucosa, and in severe cases, it can also affect the lungs, eyes, and nose, potentially spreading throughout the body. This virus leads to the multifocal necrosis of the upper respiratory tract mucosa, accompanied by extensive neutrophil infiltration and fibrin exudation and can also result in turbinate bone destruction [4].

Early symptoms of FVR include depression, sneezing, anorexia, and fever, followed rapidly by serous ocular and nasal discharge, potentially accompanied by bilateral conjunctivitis, conjunctival edema, and hyperemia. As the disease progresses, the ocular and nasal discharge may become purulent, with crust formation around the nostrils and eyelids. In severe cases, respiratory distress and coughing may occur, potentially leading to viral pneumonia [5]. Non-immunized kittens under three months of age often experience secondary bacterial infections after FeHV-1 infection, resulting in a high mortality rate [6]. Following primary FeHV-1 infection, over 80% of cats remain persistently infected, with potential intermittent viral shedding occurring one or more times [7]. Around 45% of these cats may experience viral reactivation due to stress, illness, or other environmental factors [8], and approximately 70% may experience reactivation after corticosteroid administration [7]. FeHV-1 can be isolated from the cornea, nasal turbinates, olfactory bulb, brain, optic nerve, and trigeminal ganglion (TG), during both the acute and latent phases of infection [9]. Even cats vaccinated against FeHV-1 remain at risk of infection, as intramuscular or intranasal vaccines can only partially suppress the clinical symptoms and do not prevent viral reactivation [10].

Currently, the clinical treatment of FVR primarily includes antiviral therapy and supportive care. However, the effectiveness of existing antiviral drugs against FeHV-1 is often limited, highlighting the need for alternative therapeutic strategies. Recent studies have explored various natural and synthetic compounds with potential antiviral activity. For example, Nitazoxanide, miltefosine, green tea extract, and natural compounds such as Saikosaponin B2, Punicalin, and Punicalagin have demonstrated inhibitory effects on FeHV-1 through mechanisms like viral entry blockade, immune modulation, and oxidative stress reduction. These findings provide valuable insights into developing more effective and safer treatments for FeHV-1 infections [11,12,13].

*Artemisia argyi*, a herb with a long history of use in traditional Chinese medicine, not only holds an important place in traditional medicine but has also demonstrated multiple biological activities validated by modern science. The chemical composition of *Artemisia argyi* primarily includes essential oils, flavonoids, and terpenoids, which endow it with various pharmacological effects, including antioxidant, anti-inflammatory, antibacterial, and antiviral properties [14,15,16,17]. Among these, essential oil components such as eucalyptol, α-thujone, camphor, and borneol have shown prominent therapeutic effects against respiratory viral infections and possess favorable pharmacokinetic properties [18,19]. Essential oils can be rapidly absorbed and distributed to key organs, including the lungs [20,21]. The essential oil of *Artemisia argyi* has significant respiratory anti-inflammatory and antioxidant properties [22], as well as antiviral properties [23]. This study thus focuses on *Artemisia argyi* essential oil (AAEO) to explore its molecular mechanisms in intervening in FeHV-1 infection.

## 2. Materials

### 2.1. Experimental Animals

Thirty-five unvaccinated, healthy domestic shorthair cats, aged 2–3 months, with a gender ratio of 1:1, were purchased from a cattery with no history of FVR. Deep anesthesia was induced by intravenously injecting propofol at a dose of 1.5 mg/kg, followed by euthanasia via the intravenous injection of potassium chloride at a dose of 75 mg/kg. This experiment was approved by the Institutional Animal Care and Use Committee of China Agricultural University (Ethics Approval No.: AW22104202-2-2).

### 2.2. Extraction of Artemisia argyi Essential Oil (AAEO)

The dried leaves of *Artemisia argyi* were purchased from Beijing Tongrentang Co., Ltd. (Beijing, China). The volatile oil from the leaves was extracted using the water extraction coupling rectification (WER) technique [24,25,26]. The extraction process yielded an average volatile oil content of 0.53%, with a mass concentration of 0.9507 g/mL.

The volatile oil was metabolically analyzed using liquid chromatography–mass spectrometry (LC-MS) and gas chromatography–mass spectrometry (GC-MS) platforms. The top 50 metabolites identified by the LC-MS and GC-MS platforms are shown in Table 1.

### 2.3. Cells

Feline kidney cells (F81) were provided and preserved by the Traditional Chinese Veterinary Medicine Teaching Laboratory at China Agricultural University. The cells were cultured in DMEM supplemented with 10% fetal bovine serum (FBS) and 1% antibiotics. The cultures were maintained at 37 °C in a humidified incubator with 5% CO_2_.

### 2.4. Virus

The FeHV-1 ZN09 strain was provided by the Traditional Chinese Veterinary Medicine Teaching Laboratory at China Agricultural University. The feline herpesvirus-1 (FeHV-1) strain used in this study was isolated from a naturally infected cat showing clinical symptoms of infection. The TCID_50_ of the FeHV-1 ZN09 strain in F81 cells was measured to be 10^−5.1^/0.1 mL.

## 3. Methods

### 3.1. Modeling and Treatment Procedure

Thirty-five cats, aged 2–3 months and screened to be FHV antibody-negative, were randomly divided into five groups: a blank control group, model group (Group FHV), high-dose AAEO treatment group (Group High), medium-dose AAEO treatment group (Group Medium), and low-dose AAEO treatment group (Group Low). Each group contained 7 cats with a male-to-female ratio (3:4 or 4:3). The cats were housed in their allocated groups and adaptively housed for 2 weeks to alleviate stress from transportation. During this period, they underwent skin and fecal examinations and routine deworming, and they had free access to food and water. The group housing environment was maintained at a temperature of 25 ± 2 °C and a relative humidity of 50 ± 5%, monitored daily to ensure consistency.

To establish the FeHV-1 infection model, 0.5 mL of FeHV-1 ZN09 viral cell culture medium was administered intranasally to the FHV group and the three AAEO treatment groups, while the blank control group received 0.3 mL of serum-free DMEM cell culture medium intranasally. At 72 h post-inoculation, treatment commenced. The three AAEO treatment groups received intranasal administrations of 0.6%, 0.3%, and 0.15% AAEO solutions, respectively, twice daily, for 7 days. The blank control and model groups received 0.4% normal saline intranasally, with the treatment period starting on day 1 (D1).

Daily observations were made to record the mental state, clinical symptoms, body temperature changes (rectal temperature), weight changes, and mortality of each group. Clinical symptom scores were assigned and recorded in detail according to the FeHV-1 clinical symptom scoring standard [27] (Table 2). Tissue samples were collected on day 7.

### 3.2. Collection and Processing of Tissue Samples

Samples from the conjunctiva (both eyes), throat, trachea (three segments), lungs (2 mm × 2 mm × 2 mm samples from each lobe), and olfactory bulb were collected from all cats. All samples were placed in sterile EP tubes and stored at −80 °C for future use.

The turbinate bone, trachea, and lungs were fixed in 4% paraformaldehyde, with the turbinate bone requiring decalcification after fixation.

Nasal mucosa tissues were thoroughly frozen in liquid nitrogen for subsequent transcriptomic and proteomic analysis.

### 3.3. Histology Analysis

The tissue samples fixed in paraformaldehyde were cut into 3 × 3 × 5 mm blocks and placed in embedding cassettes. The samples were dehydrated using a graded ethanol series (50%, 70%, 80%, 90%, 100%, and 100%, each for 1 h). Following dehydration, the samples were cleared in xylene (1:1 xylene and alcohol for 30 min, followed by pure xylene I and xylene II for 10 min each). The cleared tissues were then infiltrated with paraffin and embedded.

The paraffin blocks were frozen overnight at −20 °C. The following day, the blocks were sectioned into 4 μm thick slices using a manual rotary microtome (Leica RM2235, Leica Biosystems, Shanghai, China). The sections were mounted on glass slides and subsequently stained with hematoxylin and eosin (HE) for histological examination. The slides were then visualized under a light microscope (OLYMPUS CX21, Olympus Corporation, Tokyo, Japan).

### 3.4. Nucleic Acid Extraction and FHV Viral Load Detection

Nucleic acids from tissues such as the throat, conjunctiva, trachea, and lungs were extracted using the MagBio Tissue DNA/RNA Kit (Zhuhai Baotai Instrument Biotechnology Co., Ltd., Zhuhai, China). Approximately 120 mg of tissue sample was weighed and placed into a 2 mL sterile EP tube. Subsequently, 1.5 mL of pre-treatment solution and 20 μL of proteinase K were added. The mixture was homogenized using an automated rapid sample grinder (Beijing Diatex Bioscience Co., Ltd., Beijing, China), following the parameters listed in Table 3, until no visible tissue fragments remained. The homogenized mixture was vortexed and incubated in a 55 °C water bath for 60 min, with vortexing for 5–10 s every 30 min. The mixture was then centrifuged at 12,000 rpm for 3 min, and the supernatant was collected as the extraction sample. The processed samples were loaded into a 96-well plate at 200 μL per well and processed using an automatic nucleic acid extractor (BTE-32) (Zhuhai Baotai Instrument Biotechnology Co., Ltd.) following the protocol outlined in Table 4. The extracted nucleic acids were stored at −20 °C.

The viral load of FHV in the throat, conjunctiva, proximal trachea, distal trachea, lungs, and olfactory bulb was detected using the probe method. The primer sequences, synthesized by Sangon Biotech (Shanghai) Co., Ltd., Shanghai, China, are listed in Table 5. The reaction procedure was as follows: initial denaturation at 95 °C for 60 s, followed by 40 cycles of 95 °C for 20 s and 60 °C for 60 s. The relative viral copy number was determined based on the reaction CT values.

### 3.5. Cytotoxic Assay of AAEO on F81 Cells

Preparation of *Artemisia argyi* essential oil (AAEO) solution: Freshly prepare the solution as needed. Mix 20 μL of AAEO with 30 μL of dimethyl sulfoxide (DMSO) (2:3 ratio) and vortex thoroughly to ensure complete mixing. Then, add the mixture to a DMEM cell culture medium containing 10% fetal bovine serum to dilute it to a stock solution of 380 μg/mL (with a final DMSO concentration of less than 0.1%). Further, dilute this stock solution to the desired experimental concentrations.

Cell Counting Kit-8 (CCK-8) Assay: Prepare a cell suspension from 10 mL of confluent F81 cells and plate 100 μL of the cell suspension per well in a 96-well plate, setting up six replicates for each condition. Incubate the plate at 37 °C with 5% CO_2_ for 6 h to allow the cells to adhere. After adhesion, discard the culture medium and add 100 μL containing AAEO at 47.5, 95.0, 142.5, 190.0, 237.5, 285.0, and 332.5 μg/mL to the respective wells. Set up a blank control group and a culture medium control group. After incubating the plates for 6, 12, and 18 h, add 100 μL of PBS solution containing 10% CCK-8 to each well. Incubate the plates in the incubator for 1 h, then shake them on a microplate reader for 1 min and measure the absorbance (A) at 450 nm. Calculate the survival rate of F81 cells and determine the half-maximal inhibitory concentration (IC_50_) to indicate the cytotoxicity of *Artemisia argyi* essential oil.

Cell viability = [(As − Ab)/(Ac − Ab)] × 100%

As: Absorbance A of the experimental wells with AAEO (with different concentrations of AAEO);
Ac: Absorbance of the control wells (without the drug);
Ab: Absorbance of the blank wells (without cells and drugs).

### 3.6. In Vitro Anti-FeHV-1 Assay of AAEO

Dilution of *Artemisia argyi* Essential Oil (AAEO): Following the method described in Section 3.5, a stock solution of AAEO was prepared. This stock solution was then gradually diluted using DMEM containing 2% fetal bovine serum to obtain AAEO dilutions at concentrations of 38.0 μg/mL, 76.0 μg/mL, 126.7 μg/mL, 152.0 μg/mL, and 190.0 μg/mL. The experiment included a cell blank control group, a virus control group, and AAEO treatment groups at different concentrations, with four replicates for each group. The AAEO was administered according to the following three treatment protocols:

Protective Effect on Cells (Pre-treatment with AAEO): A 96-well plate with confluent monolayers of F81 cells was used. Different concentrations of AAEO dilution (100 μL per well) were added to the respective treatment groups. The plates were incubated at 37 °C with 5% CO_2_ for 90 min. After incubation, the cells were washed twice with serum-free DMEM. Then, 100 μL of virus solution containing 100 TCID_50_ of FeHV-1 was added to the virus control group and the AAEO treatment groups, followed by another 90 min incubation under the same conditions. After washing twice with serum-free DMEM, the medium was replaced with DMEM containing 2% fetal bovine serum. The cell blank control group received no further treatment. After 24 h of incubation at 37 °C with 5% CO_2_, the OD450 was measured using the CCK-8 assay as described in Section 3.5.

Direct Effect on Virus (Simultaneous Addition of AAEO and Virus): Different concentrations of AAEO were mixed 1:1 with an equal volume of virus solution containing 100 TCID_50_ of FeHV-1. The mixtures were incubated for 90 min, and then 100 μL of each mixture was added to the respective wells of a 96-well plate with confluent F81 cells. The plate was incubated at 37 °C with 5% CO_2_ for 90 min. After incubation, the cells were washed twice with serum-free DMEM and replaced with DMEM containing 2% fetal bovine serum. The subsequent steps were the same as the first experiment.

Therapeutic Effect after Virus Infection (Post-treatment with AAEO): A 96-well plate with confluent F81 cells was used. A virus solution containing 100 TCID_50_ of FeHV-1 (100 μL per well) was added to the virus control and AAEO treatment groups. The plate was incubated at 37 °C with 5% CO_2_ for 90 min. After incubation, the cells were washed twice with serum-free DMEM, followed by adding 100 μL of AAEO at different concentrations to the respective treatment groups. The plates were incubated under the same conditions for an additional 90 min. After another washing step with serum-free DMEM, the medium was replaced with DMEM containing 2% fetal bovine serum. The subsequent steps were the same as those in the first experiment.

### 3.7. Transcriptomic Sequencing and Analysis

**RNA extraction library construction and RNA sequencing**

Total RNA was extracted from nasal mucosal samples using TRIzol reagent (Invitrogen, Darmstadt, Germany) according to the manufacturer’s protocol. After grinding the frozen tissue under liquid nitrogen, 100 mg of each sample was homogenized in TRIzol, and RNA was isolated using chloroform and isopropanol precipitation. The RNA was washed with 75% ethanol, air-dried, dissolved in RNase-free water, and stored at −80 °C. RNA purity and concentration were measured with a NanoDrop 2000 spectrophotometer (Thermo Scientific, Waltham, MA, USA), and RNA integrity was assessed using the Agilent 2100 Bioanalyzer (Agilent Technologies, Santa Clara, CA, USA). Libraries were constructed with the VAHTS Universal V5 RNA-seq Library Prep Kit (Vazyme Biotech, Nanjing, China) and sequenced on the Illumina Novaseq 6000 platform (Illumina, San Diego, CA, USA) to obtain 150 bp paired-end reads, generating approximately 50 million reads per sample.

Raw sequencing data were processed using fastp v0.20.1 [28]. Gene expression was quantified in fragments per kilobase of transcript per million mapped reads (FPKM) after alignment to the reference genome using HISAT2 v2.1.0 [29]. Read counts were obtained using HTSeq-count v0.11.2 [30], and differential expression analysis was performed with DESeq2 v1.22.2 and DESeq v1.34.1. Principal component analysis (PCA) was conducted in R v3.2.0 to evaluate the differences in RNA expression between sample groups.

**Functional analysis of differentially expressed genes (GO and KEGG enrichment)**

Differentially expressed genes (DEGs) were identified using DESeq2 with a threshold of *p*-value < 0.05 and fold change > 2 [31]. The hierarchical clustering of DEGs was performed in R (v 3.2.0), and the results were visualized using heatmaps and volcano plots. GO (Gene Ontology) and KEGG (Kyoto Encyclopedia of Genes and Genomes) pathway enrichment analyses were conducted using a hypergeometric distribution algorithm to identify significantly enriched functional categories. The GO analysis compared the DEGs with the annotated proteome of Felis catus (Felis catus AR104), and Fisher’s exact test was used to determine the significance of enrichment in cellular component (CC), biological process (BP), and molecular function (MF) categories (*p*-value < 0.05). The KEGG analysis identified significantly enriched biological pathways in DEGs by comparing them with the KEGG annotation of Felis catus, categorizing pathways into seven branches, including cellular processes, metabolism, and organismal systems.

### 3.8. Proteomics Sequencing and Analysis

**Proteomic analysis**

Total protein was extracted from nasal mucosal samples using a lysis buffer containing 0.1% Triton-100 and 8 M urea. After sonication and centrifugation, the proteins were reduced with TCEP and alkylated with IAA. Trypsin digestion was performed in two steps, followed by desalting using Thermo Fisher Scientific desalting columns (Thermo Fisher Scientific, Waltham, MA, USA). Peptides were fractionated using the DIONEX UltiMate 3000 system (Thermo Scientific, San Jose, CA, USA) with an XBridge Peptide BEH C18 column (Waters Corporation, Milford, MA, USA). The fractions were dried and reconstituted in a solution of 2% ACN and 0.1% formic acid for mass spectrometry analysis. The final peptide concentration for Data-Independent Acquisition (DIA) was set to 0.2 μg/μL, with a sample injection volume of 1 μL.

**Differential protein expression analysis and enrichment analysis (GO, KEGG)**

Differential expression analysis and enrichment analysis of differentially expressed proteins (DEPs) were performed using the GO and KEGG databases, following the same method as that in the transcriptomic analysis.

### 3.9. Statistical Analysis

In the cytological and clinical scoring sections, continuous variables were expressed as means ± standard deviations. Comparisons between two groups were conducted using an independent samples *t*-test, while a one-way analysis of variance (ANOVA) was used to analyze statistical significance among multiple groups. Statistical analyses were performed using SPSS software version 26.0, and the results were visualized using GraphPad Prism 9. A *p*-value < 0.05 was considered statistically significant.

In the transcriptome analysis section, the HISAT2 v2.1.0 software package was used for genome alignment, and gene expression levels (FPKM) were calculated [29]. HTSeq-count was employed to obtain the read counts for each gene [30]. In the proteomics analysis section, DIA-NN (v1.8.1) was used to process mass spectrometry data to obtain qualitative and quantitative data.

PCA and plotting were performed using R (v3.2.0). Differential expression analysis was conducted using the DESeq2 v1.22.2 software package [31], with genes or proteins meeting the criteria of q < 0.05, and a fold change > 2 or fold change < 0.5 defined as DEGs/DEPs.

## 4. Results

### 4.1. Clinical Symptoms Changes

Survival Rate Statistics: On the seventh day, all the animals in the blank group, high-dose group, and medium-dose group survived (7/7 each). In the low-dose AAEO group, one animal died (6/7), while in the FHV group, two animals died (5/7), and three were in a moribund state, resulting in a significantly lower survival outlook.

Clinical Symptoms and Scoring: At 72 h post-inoculation (designated as D1), animals in all groups, except the blank control group, exhibited common clinical symptoms, including conjunctival redness and swelling, increased serous eye discharge, and frequent sneezing, along with signs of lethargy and decreased appetite.

**Figure 1:**
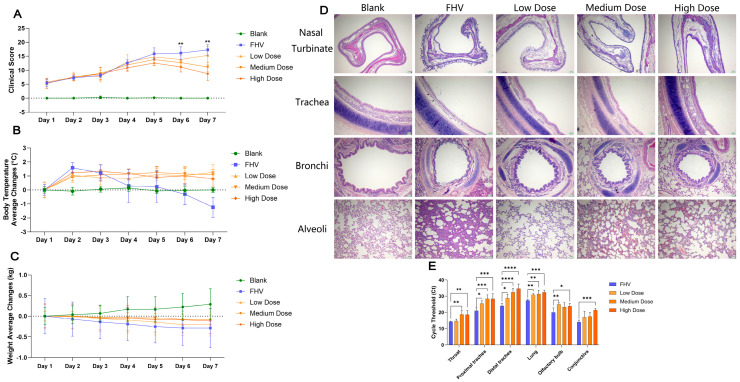
Overall physiological manifestations and pathological changes in cats. (**A**) Changes in clinical symptom scores during 7 days of treatment. The scores in the FHV group increased daily, while those in the three AAEO treatment groups began to decrease from day five. Significance levels: ** *p* < 0.01, versus medium- and high-dose groups. (**B**) Body temperature changes during 7 days of treatment. The body temperature of the three AAEO treatment groups remained consistently higher compared to the blank group, while the FHV group showed higher temperatures only during the first three days, followed by a gradual decline. (**C**) Average weight changes during 7 days of treatment. The three AAEO groups experienced less weight loss compared to the FHV group. (**D**) Pathological comparison of nasal turbinate (100×), trachea (100×), bronchi (100×), and alveoli (100×) among different groups. (**E**) Viral loads in different tissues: the Ct values in the FHV group were significantly lower across all tissues compared to those in the AAEO treatment groups, indicating higher viral loads. Significance levels: * *p* < 0.05, ** *p* < 0.01, *** *p* < 0.001, and **** *p* < 0.0001 versus the FHV group.

From the first to the fifth day, clinical symptoms of animals in the FHV group and the AAEO treatment groups progressively worsened, with clinical scores increasing over time. In the model group, starting from day 4, eye and nasal secretions became purulent, with some cases showing crust formation around the eyes and nose, leading to purulent discharge sealing the eyes shut. Although the AAEO treatment groups also showed purulent secretions, the severity was notably milder compared to that in the model group, despite a noticeable increase in sneezing frequency. Starting on the sixth day, the symptom scores in the FHV group continued to rise, while the scores in the AAEO treatment groups declined, showing significant differences (*p* < 0.05). By day 7, the model group exhibited severe symptoms such as purulent discharge from the mouth and nose and open-mouth breathing. In contrast, the AAEO treatment groups showed relief in respiratory difficulties, with no significant differences observed among the high-, medium-, and low-dose groups (Figure 1A).

Body Temperature Changes: From the first day of treatment, the rectal temperature of cats in all groups was recorded and plotted as a curve. Compared to the relatively stable temperature in the blank group, both the FHV and AAEO groups showed an increase in average temperature on the second day. The AAEO groups maintained a higher temperature level, while the temperature in the FHV group began to decrease starting on day 4 (Figure 1B).

Body Weight Changes: The average weight in the blank group showed a steady increase, while the FHV group exhibited a gradual decrease in weight. The AAEO groups showed a slight overall weight decrease, with the high- and medium-dose treatment groups maintaining a relatively stable weight. In contrast, the low-dose treatment group experienced a gradual weight decline, although a slight recovery was observed starting from day 7 (Figure 1C).

### 4.2. Histopathological Examination

Based on hematoxylin and eosin (HE) staining, the model group subjected to FeHV-1 inoculation exhibited severe detachment of the pseudostratified ciliated columnar epithelium in the nasal turbinate, trachea, and small bronchi. Significant goblet cell hyperplasia, the thickening of the basement membrane, and abnormally loose submucosal connective tissue were observed. In the treatment groups, as the concentration of AAEO increased, a reduction in goblet cell density per unit area and decreased edema in the submucosal connective tissue were noted. Notably, no significant lesions were found in the bronchioles across all treatment groups. Another key observation was that FeHV-1 infection disrupted the glandular structure in the trachea, small bronchi, and bronchioles, while AAEO treatment mitigated this damage. Moreover, the number and density of glandular structures in various tissues increased significantly with higher AAEO concentrations, with the high-concentration treatment group even surpassing the blank control group in glandular density (Figure 1D).

Nasal Turbinate: Under 100× magnification, the model group showed vascular dilation, submucosal edema, mucus gland hyperplasia, increased goblet cells, epithelial cell detachment, and inflammatory cell infiltration in the submucosa of the nasal turbinate. In the AAEO treatment groups, some epithelial cell detachment, submucosal edema, and irregular arrangement of columnar cells were observed. With increasing AAEO concentrations, goblet cell hyperplasia tended to be atypical.

Trachea: At 100× magnification, the blank control group displayed intact ciliary structures, densely packed columnar cells arranged in an orderly manner, and sparsely distributed goblet cells among the columnar cells. The basement membrane, composed of basal cells, was dense and simple in structure. The submucosal layer was rich in glandular structures with large glandular lumens, most of which were in a resting state. In contrast, the model group exhibited significant detachment of cilia and columnar epithelial cells, with the basement membrane directly exposed to the airway and showing noticeable thickening. The remaining goblet cells showed marked hyperplasia. The submucosal connective tissue was edematous, and the glandular structures were severely reduced in number and extensively damaged. In the AAEO treatment groups, the ciliary and columnar epithelial cell structures remained largely intact, and the goblet cell density decreased with increasing AAEO concentrations, although it was still significantly higher than in the blank control group. The basement membrane was slightly thinner than in the model group but still thicker than in the blank control group. The degree of submucosal edema decreased with higher AAEO concentrations, and the number of glandular structures increased, with all glands in a hypersecretory state, containing abundant mucus.

Bronchi and Bronchioles: At 100× magnification, the changes in the bronchial and bronchiolar mucosal layers, as well as the glandular structures, in all groups generally mirrored the trends observed in the tracheal mucosal layer.

Alveoli: Under 100× magnification, the alveolar walls in the blank control group appeared thin, with thin layers of connective tissue separating adjacent alveoli, containing a continuous capillary network and abundant elastic fibers. In the model group, type I alveolar cell damage, type II alveolar cell hyperplasia, fibrous tissue proliferation, widened alveolar septa, and alveolar fusion and expansion were observed. Low-dose AAEO treatment showed some improvement in alleviating alveolar wall hyperplasia, but in the medium- and high-dose groups, emphysematous changes and congestion within the bronchial walls were noted.

### 4.3. Viral Load

The experimental results shown in Figure 1E indicate that the Ct values in various tissues of the FHV group are significantly lower than those in the treatment groups (*p* < 0.05), indicating a relatively higher viral load. There is no significant difference in viral load between the different AAEO treatment groups. Additionally, it can be observed that as the virus progresses deeper into the cat’s respiratory tract, the overall viral load shows a decreasing trend.

### 4.4. Cytotoxicity of AAEO on F81 Cells

**Figure 2:**
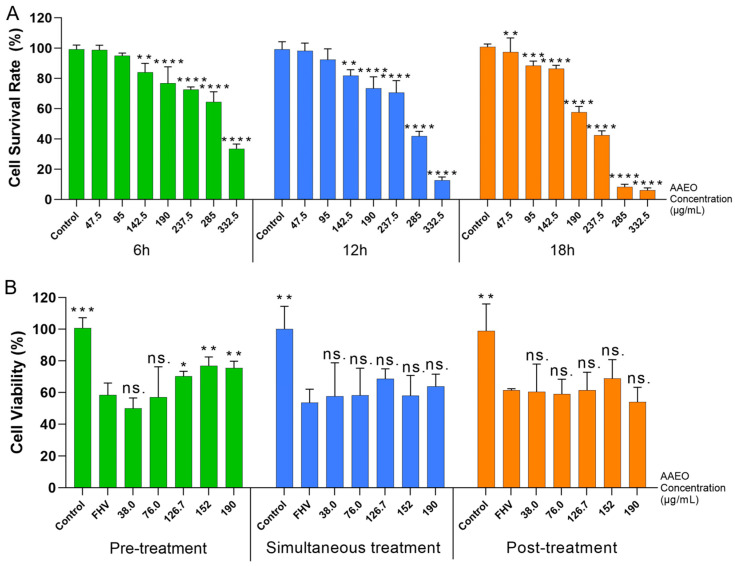
Cytotoxicity and antiviral effect of AAEO in vitro. (**A**) Effects of different concentrations of AAEO on the cell survival rate of F81 cells. AAEO significantly inhibited the survival rate of F81 cells at concentrations higher than 190 μg/mL. Significance levels: ** *p* < 0.01, *** *p* < 0.001, and **** *p* < 0.0001, versus the control group. (**B**) In vitro anti-FeHV-1 activity of AAEO with different administration methods. Only the pre-treatment group demonstrated a protective effect on F81 cells. Significance levels: ns. Not significant, * *p* < 0.05, ** *p* < 0.01, and *** *p* < 0.001, versus the FHV group.

Five different concentrations of AAEO, ranging from 47.5 to 332.5 μg/mL, were used to assess the cytotoxicity toward F81 cells. The cell viability was measured at 6, 12, and 18 h post-treatment. According to the experimental results (Figure 2A), the IC_50_ of AAEO was determined to be 229.3 μg/mL (95% confidence interval: 208.9–294.4 μg/mL). As shown in Figure 2A, after 18 h of treatment with AAEO, if the concentration exceeded 285 μg/mL, the growth inhibition rate of F81 cells surpassed 90%.

### 4.5. In Vitro Anti-FeHV-1 Activity of AAEO

In the experiment, five concentrations of AAEO ranging from 38.0 to 190.0 μg/mL were selected and administered using three different treatment methods. After 12 h of treatment, the relative viability of F81 cells infected with FeHV-1 was measured and calculated. The experimental results are shown in Figure 2B.

In Figure 2B, it can be observed that in the pre-treatment method, the relative cell viability in the 126.7 μg/mL AAEO treatment group showed a statistically significant difference compared to the virus control group. Moreover, AAEO concentrations in the range of 152 to 190 μg/mL exhibited even more pronounced statistical differences. In the other two groups, it can be noted that the relative cell viability in the AAEO treatment groups, across the concentration range of 38 to 190 μg/mL, did not show statistically significant differences compared to the FHV group.

### 4.6. Transcriptomic Analysis

#### 4.6.1. RNA Sequencing and Differential Gene Expression Analysis

**Figure 3:**
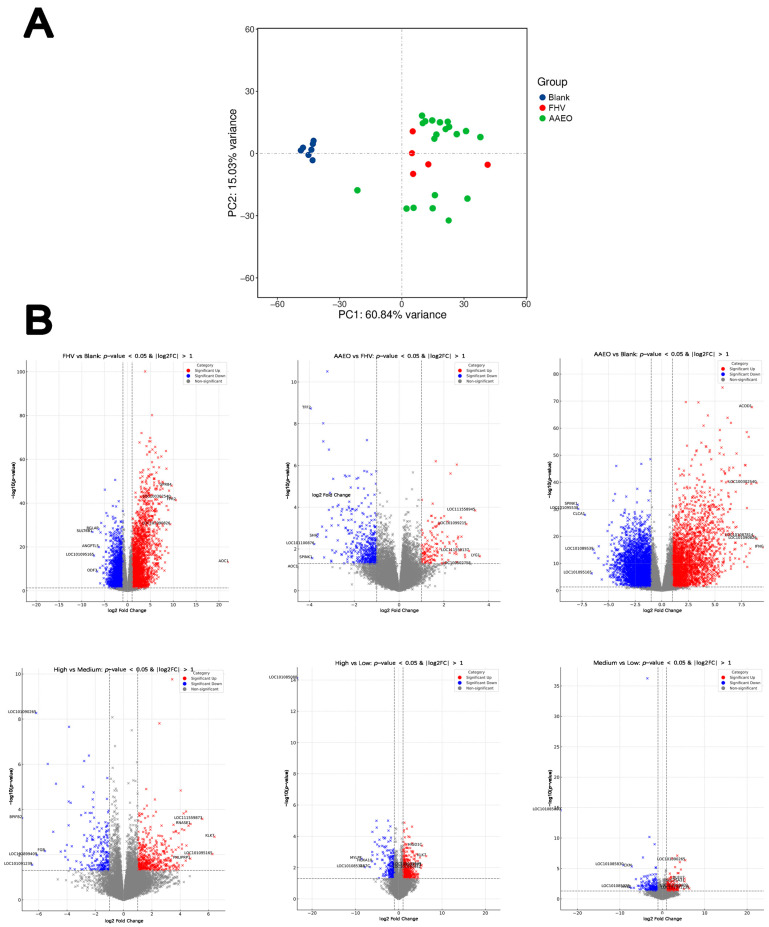
Noteworthy transcriptomic analysis results. (**A**) Principal component analysis showing the overall separation of the blank and the other groups based on the RNA-sequencing profiles. (**B**) Volcano plots showing differentially upregulated genes (red dots) and downregulated genes (blue dots) due to FHV infection and AAEO treatment (FDR < 0.05). Non-significant genes are shown in gray dots.

A total of 19,273 expressed transcripts were obtained from the nasal mucosal samples of cats through RNA-seq expression profiling. Of these, only those annotated with a known gene symbol were included in the gene set enrichment analyses. As shown in Figure 3A, the PCA demonstrated a clear separation between the blank control and FHV and treatment groups in terms of overall gene expression patterns, with the first principal component (PC1) explaining 60.84% of the total variance. While the treatment groups exhibit some dispersion along the second principal component (PC2, 15.03% variance), the separation between FHV and treatment groups is minimal (Figure 3A).

#### 4.6.2. Transcriptomic Response of Cat Nasal Mucosa to FHV Infection and AAEO Treatment

When comparing the FHV group with the control group, a total of 2533 genes were significantly upregulated, and 3229 genes were significantly downregulated in the nasal mucosa tissue samples. The most significantly upregulated genes included Amiloride-sensitive amine oxidase (AOC1, log2FC = 22.13), N-formyl peptide receptor (PR2, log2FC = 10.59), and G protein-coupled receptor 84 (GPR84, log2FC = 9.65). Among the most significantly downregulated genes were Sulfotransferase 6B1 (SULT6B1, log2FC = −7.6), Sulfotransferase 1C1 (LOC101095165, log2FC = −7.32), and Outer dense fiber protein 3 (ODF3, log2FC = −6.6). In the nasal mucosa samples of the three treatment groups, compared to the control group, 2595 genes were significantly upregulated, and 3859 genes were significantly downregulated, resulting in a total of 6454 DEGs. The most significantly upregulated genes included Interferon gamma (IFNG, log2FC = 9.57), Peptidoglycan recognition protein 4 (LOC101090826, log2FC = 8.91), and C-C motif chemokine 3 (LOC100302540, log2FC = 8.90). The most significantly downregulated genes included Serine protease inhibitor Kazal-type 1 (SPINK1, log2FC = −7.91), Zymogen granule membrane protein 16 (LOC101095538, log2FC = −7.85), and Sulfotransferase 1C1 (LOC101095165, log2FC = −6.58). When comparing the three treatment groups to the FHV group, 49 genes showed significant changes, with 6 genes upregulated and 43 genes downregulated. The most significantly upregulated genes were Phospholipid transfer protein (LOC111558945, log2FC = 3.39), DLA class II histocompatibility antigen, DR-1 beta chain (LOC111556169, log2FC = 2.57), and LINE-1 retrotransposable element ORF2 protein (LOC102901712, log_2_FC = 2.29). Among the most significantly downregulated genes were Trefoil factor 2 (TFF2, log2FC = −3.39), Trefoil factor 3 (TFF3, log2FC = −3.38), and Bactericidal permeability-increasing protein (BPI, log2FC = −3.38) (Figure 3B).

#### 4.6.3. Gene Enrichment Analysis

GO Analysis

**Figure 4:**
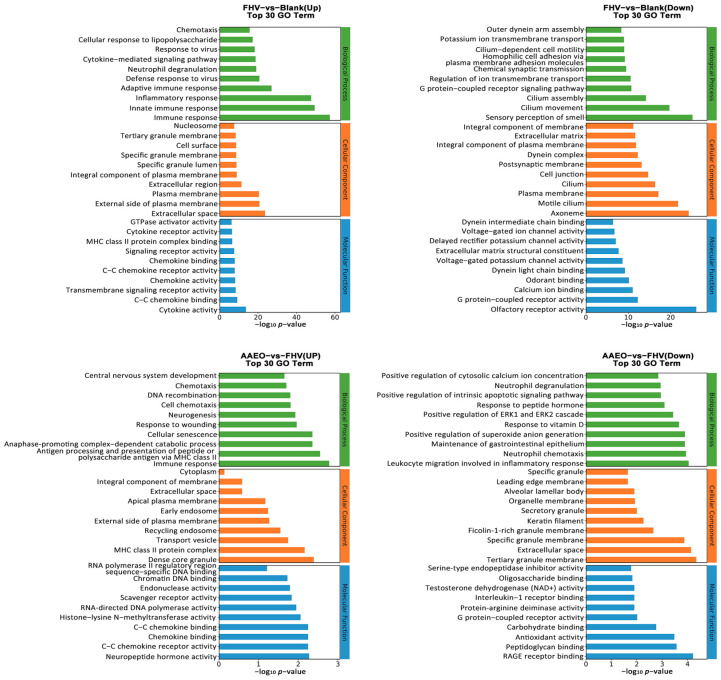
Transcriptomic GO enrichment analysis. GO enrichment analysis identified GO terms with PopHits ≥ 5 in the upregulated and downregulated BP, CC, and MF categories for comparisons between the blank and FHV groups, and FHV and AAEO groups. The top 10 GO terms in each category were ranked by −log10 *p*-value. The vertical axis shows the GO term names, and the horizontal axis represents the −log10 *p*-value.

Compared with the blank group, the GO enrichment analysis of the FHV group showed significant upregulation in immune-related BPs. The main enriched items included immune response, innate immune response, inflammatory response, and response to viruses. In contrast, BPs related to cilium-dependent cell motility, outer dynein arm assembly, and potassium ion transmembrane transport were significantly downregulated. CCs were enriched in the plasma membrane, extracellular matrix, and cell surface, while downregulated components included cilium, dynein complex, and cell junction. In terms of MF, cytokine activity, chemokine binding, and G protein-coupled receptor activity were significantly upregulated, while voltage-gated ion channel activity and calcium ion binding were significantly downregulated (Figure 4).

Compared with the FHV group, the GO enrichment analysis of the three AAEO treatment groups showed that, in terms of the BPs, immune response, antigen processing, the presentation of peptide or polysaccharide antigen via MHC class II, and anaphase-promoting complex-dependent catabolic process were the most significantly upregulated. Meanwhile, leukocyte migration involved in the inflammatory response, neutrophil chemotaxis, and the maintenance of gastrointestinal epithelium were the most significantly downregulated. Regarding the CC category, the upregulated parts included central nervous system development, dense core granule, and MHC class II protein complex, while the downregulated parts included tertiary granule membrane, extracellular space, and specific granule membrane. In terms of MF, neuropeptide hormone activity, C-C chemokine receptor activity, and chemokine binding were the top three upregulated functions, while RAGE receptor binding, peptidoglycan binding, and antioxidant activity were the top three downregulated functions (Figure 4).

KEGG Analysis

**Figure 5:**
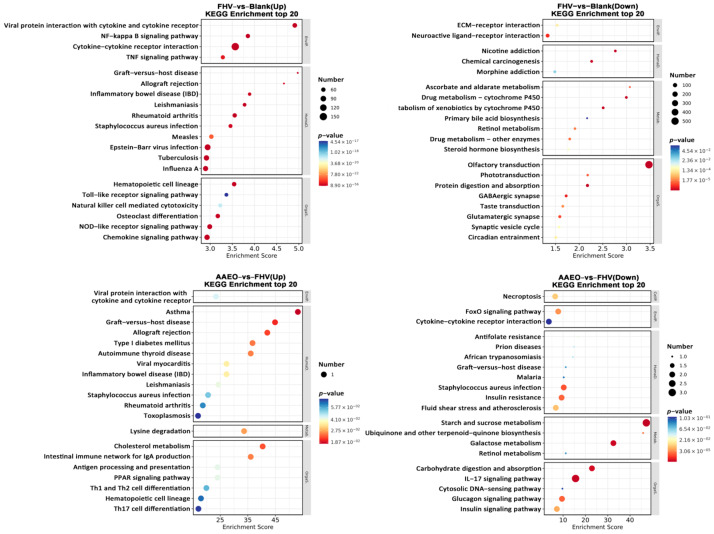
Transcriptomic KEGG enrichment analysis. KEGG enrichment analysis identified upregulated and downregulated pathways with PopHits ≥ 5 for comparisons between the blank and FHV groups, and FHV and AAEO groups. The pathways were ranked in descending order based on their −log10 *p*-values. The horizontal axis represents the Enrichment Score, with larger bubbles indicating a higher number of differentially expressed protein-coding genes in the pathway. Bubble colors range from blue to white to yellow to red, with smaller *p*-values indicating higher significance.

In the KEGG enrichment analysis, compared with the blank group, the FHV group showed significant upregulation of gene expression in pathways such as the NF-kappa B signaling, TNF signaling, and NOD-like receptor Tsignaling pathways, while genes related to olfactory transduction were significantly downregulated. In the AAEO treatment groups compared with the FHV group, genes related to asthma and cholesterol metabolism were significantly upregulated, while genes related to starch and sucrose metabolism and the IL-17 signaling pathway were significantly downregulated (Figure 5).

### 4.7. Proteomics Analysis

#### 4.7.1. PCA

**Figure 6:**
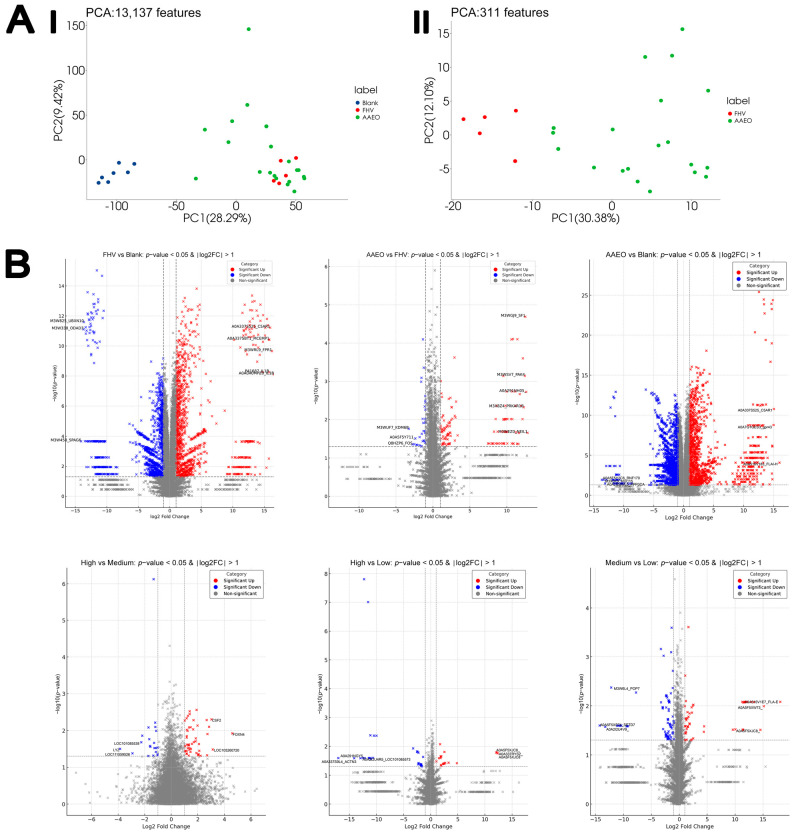
Noteworthy proteomics analysis results. (**AI**,**AII**) The principal component analysis based on mass spectrometry not only revealed the overall separation among the blank, FHV, and AAEO treatment groups but also showed a significant separation between the FHV and AAEO groups when compared directly. (**B**) Volcano plots showing differentially upregulated proteins (red dots) and downregulated proteins (blue dots) due to FHV infection and AAEO treatment (FDR < 0.05). Non-significant genes are shown in gray dots.

In the PCA of overall differential proteins (Figure 6(AI)), a significant difference in gene expression patterns between the FHV group (red), the AAEO group (green), and the blank group (blue) is shown. The blank group forms a relatively independent cluster, with a clear separation from both the FHV and AAEO groups, indicating a substantial difference in overall protein expression profiles between the blank group and the FHV and AAEO treatments. After further filtering and removing proteins with a similar expression to the blank group, the remaining 331 differential proteins were used for a second PCA (Figure 6(AII)). In this analysis, the separation between the FHV and AAEO groups increased significantly, especially along PC1, showing a more pronounced difference. This suggests that while the FHV and AAEO groups exhibit some similarity in the analysis with all proteins included, the removal of proteins with a similar expression to the blank group amplifies the difference between the FHV and AAEO groups, showing a more distinct separation trend.

#### 4.7.2. Differential Protein Expression in the Nasal Mucosa of Cats in Response to FHV Infection and AAEO Treatment

In the analysis of protein expression in the FHV infection group compared to the blank control group, a total of 3910 DEPs were identified, including 1700 upregulated proteins and 2210 downregulated proteins. The most significantly upregulated proteins included IL1B (log2FC = 16.55), FPR1 (log2FC = 16.23), and C5AR1 (log2FC = 15.94). The most significantly downregulated proteins included SPAG6 (log2FC = −14.07), ODAD3 (log2FC = −13.68), and UBXN10 (log2FC = −13.64).

In the comparison of protein expression between the AAEO treatment groups and the FHV infection group, a total of 311 DEPs were identified, with 183 proteins upregulated and 128 proteins downregulated. The most significantly upregulated proteins included NEIL1 (log2FC = 12.80), A0A291NH05 (log2FC = 12.62), and SF1 (log2FC = 12.50). The most significantly downregulated proteins included KDM6B (log2FC = −3.27), FOS (log2FC = −2.75), and A0A5F5Y711 (log2FC = −2.37).

In the comparison between the AAEO treatment groups and the blank control group, a total of 3829 DEPs were found, with 1661 proteins upregulated and 2168 proteins downregulated. The most significantly upregulated proteins included FLAI-H (log2FC = 15.98), C5AR1 (log2FC = 15.53), and GPR43 (log2FC = 15.16). The most significantly downregulated proteins included RNF170 (log2FC = −13.65), MUC15 (log2FC = −13.37), and PPP3CA (log2FC = −13.12).

In the comparison among the high-, medium-, and low-dose AAEO treatment groups, multiple significant DEPs were identified. In the high-dose vs. low-dose comparison, a total of 69 DEPs were found, with 23 upregulated and 46 downregulated. The most significantly upregulated proteins included A0A5F5XJC8 (log2FC = 12.34), A0A337RYS2 (log2FC = 12.21), and KCNJ15 (log2FC = 12.05). The most significantly downregulated proteins included ACTN3 (log2FC = −17.05), A0A291NGY5 (log2FC = −14.13), and LOC101085573 (log2FC = −12.97). In the high-dose vs. medium-dose comparison, a total of 106 DEPs were identified, with 36 upregulated and 70 downregulated. The most significantly upregulated proteins included FOXN4 (log2FC = 4.59), LOC105260720 (log2FC = 3.12), and CSF2 (log2FC = 3.03). The most significantly downregulated proteins included LYZ (log2FC = −3.86), LOC111559526 (log2FC = −2.93), and LOC101085538 (log2FC = −2.26). In the medium-dose vs. low-dose comparison, a total of 194 DEPs were identified, with 66 upregulated and 128 downregulated. The most significantly upregulated proteins included FLA-E (log2FC = 18.07), A0A5F5XW73 (log2FC = 15.11), and A0A5F5XJC8 (log2FC = 14.53). The most significantly downregulated proteins included SETD7 (log2FC = −14.27), A0A2I2U4V9 (log2FC = −13.26), and POP7 (log2FC = −12.20) (Figure 6B).

#### 4.7.3. Protein Enrichment Analysis

GO Analysis

**Figure 7:**
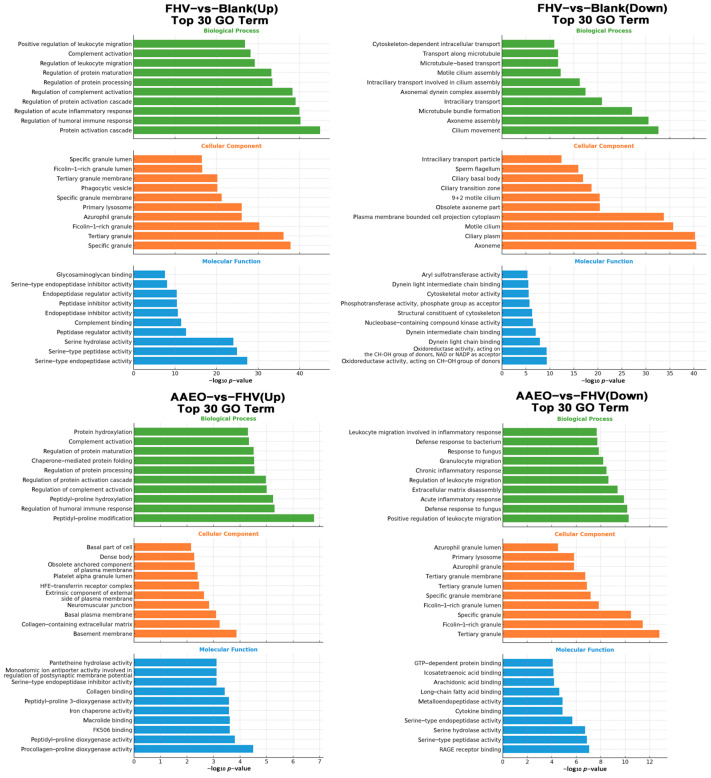
Proteomic GO enrichment analysis. The GO enrichment analysis identified GO terms with PopHits ≥ 5 in the upregulated and downregulated BP, CC, and MF categories for comparisons between the blank and FHV groups, and FHV and AAEO groups. The top 10 GO terms in each category were ranked by −log10 *p*-value. The vertical axis shows the GO term names, and the horizontal axis represents the −log10 *p*-value.

Compared with the blank group, the FHV group showed the most significant upregulation in BPs related to protein activation cascade, the regulation of humoral immune response, and the regulation of acute inflammatory response, while processes such as cilium movement, axoneme assembly, and microtubule bundle formation were significantly downregulated. In the CC category, the most significantly upregulated items were specific granule, tertiary granule, and ficolin-1-rich granule, while axoneme, ciliary plasm, and motile cilium were the most significantly downregulated. For MF, serine-type endopeptidase activity, serine-type peptidase activity, and serine hydrolase activity were the most significantly upregulated functions, whereas dynein light chain binding, oxidoreductase activity acting on the CH-OH group of donors with NAD or NADP as acceptors, and oxidoreductase activity acting on the CH-OH group of donors were the most significantly downregulated (Figure 7).

In the comparison between the AAEO treatment group and the FHV group, the most significantly upregulated BPs included peptidyl-proline hydroxylation, the regulation of humoral immune response, and peptidyl-proline modification, while the most significantly downregulated were acute inflammatory response, defense response to fungus, and the positive regulation of leukocyte migration. For the CC, the top three upregulated items were basal plasma membrane, collagen-containing extracellular matrix, and basement membrane, whereas the most significantly downregulated items were specific granule, ficolin-1-rich granule, and tertiary granule. In the MF category, the most significantly upregulated activities were FK506 binding, peptidyl-proline dioxygenase activity, and procollagen-proline dioxygenase activity, while serine hydrolase activity, serine-type peptidase activity, and RAGE receptor binding were the most significantly downregulated functions (Figure 7).

KEGG Analysis

**Figure 8:**
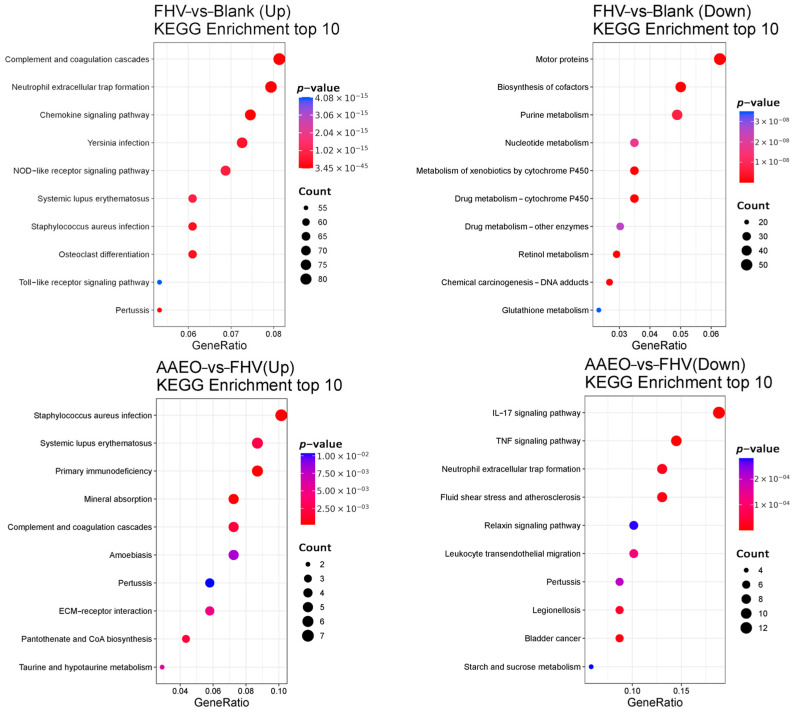
Proteomic KEGG enrichment analysis. The KEGG enrichment analysis identified upregulated and downregulated pathways with PopHits ≥ 5 for comparisons between the blank and FHV groups, and between the FHV and AAEO groups, ranked in descending order by −log10 *p*-value. The horizontal axis represents the proportion of enriched proteins within each pathway, the bubble size indicates the number of proteins enriched in the pathway, and the bubble color transitions from blue to purple to red, with smaller *p*-values indicating greater significance.

The enrichment analysis of metabolic pathways through the KEGG database revealed that compared with the blank group, the upregulated proteins in the FHV group were significantly enriched in the complement and coagulation cascades, chemokine signaling pathway, and NOD-like receptor signaling pathway. The downregulated proteins were mainly enriched in pathways related to motor proteins, the biosynthesis of cofactors, and purine metabolism (Figure 8).

In the AAEO treatment groups compared to the FHV group, the upregulated proteins were primarily enriched in pathways such as Staphylococcus aureus infection, taurine and hypotaurine metabolism, and pantothenate and CoA biosynthesis. The downregulated proteins were mainly enriched in the IL-17 signaling pathway and TNF signaling pathway (Figure 8).

## 5. Discussion

The clinical symptoms and scoring results indicate that AAEO significantly alleviates the clinical symptoms caused by FeHV-1 infection and reduces mortality to some extent. In the FHV group, a sharp drop in body temperature was observed on the sixth and seventh days of treatment, likely related to hypothermia preceding death in the experimental animals. Overall, the AAEO-treated groups maintained relatively higher and more stable body temperatures, suggesting that AAEO could control inflammation within a certain range. Weight changes showed that although the overall weight of the three AAEO groups slightly decreased, the degree of weight loss was less severe compared to the FHV group. This suggests that AAEO may help restore appetite in infected cats to some extent. Pathological examinations demonstrated that AAEO has a reparative effect on tissue damage caused by FeHV-1 infection, particularly in nasal turbinates, trachea, alveoli, and small bronchi. These effects were most pronounced in the high-dose group. However, it should also be noted that high concentrations of AAEO might cause alveolar expansion, necessitating further investigation into its mechanisms of action in lung tissue. The viral load results showed that AAEO reduces the viral load within a specific concentration range. However, its mechanisms and safety need further study.

To explore how AAEO inhibits FeHV-1 in infected cells, we conducted toxicity tests and in vitro antiviral assays on F81 with different treatment regimens. According to the results in Section 4.4, the IC_50_ of AAEO was 229.3 μg/mL (confidence interval: 208.9–294.4). This indicates that AAEO inhibits F81 cell growth to some extent, with an inhibitory effect positively correlated with its concentration. For F81 cells treated with AAEO under different regimens, only the pre-treatment group showed a significant increase in cell viability at AAEO concentrations between 126.7 and 190 μg/mL. This suggests that pre-treatment with AAEO at this concentration can mitigate subsequent viral infection-induced cellular damage. In other groups, no statistically significant differences were observed, demonstrating that AAEO does not directly inhibit the virus nor effectively treat damage caused by viral invasion. This differs significantly from the results of animal experiments, suggesting that AAEO may exert more pronounced antiviral effects in vivo by mobilizing the host immune system. These results indicate that AAEO’s antiviral activity depends not only on its direct protective effects on cells but also involves complex immune regulatory processes.

Based on the GO and KEGG analysis diagrams of gene expression and proteomics, we identified intersecting enriched terms across groups and conducted in-depth analyses of these overlapping terms. This intersection analysis reveals commonly regulated pathways at both gene expression and protein expression levels, providing better insights into how AAEO influences the pathogenic process of FHV.

For the upregulated terms in FHV infection, we identified three main categories enriched at both levels of analysis: “immune response and complement system activation”, “inflammatory response”, and “cellular granules and lysosomal activity”. The activation of the complement system plays a vital role in FHV infection as part of the innate immune defense in cats. Complement activation quickly recognizes and clears pathogens while attracting leukocytes to the infection site, enhancing local immune responses [32,33]. The common upregulation of this process at both gene and protein levels indicates the significant activation of this immune pathway, which may serve as an essential defense mechanism against FHV infection. The acute inflammatory response is crucial during the early stages of viral infection. This process regulates the release of a series of immune factors, creating an antiviral environment. The concurrent upregulation of inflammatory response aids in virus clearance but may also lead to tissue damage [34,35], one of the pathological characteristics of FHV infection [36,37]. Cellular granules and lysosomal structures in immune cells (e.g., neutrophils) store and release antimicrobial molecules. FHV infection activates these granule-related structures, which contain digestive enzymes and antimicrobial proteins that can directly kill viruses or infected cells [38,39]. This indicates that FHV infection triggers mechanisms for immune cell granule release, which plays a crucial role in defense against viral infections.

For the downregulated terms in FHV infection, several key categories were identified, including “cilium movement and assembly”, “cytoskeleton-dependent transport”, and “dynein complexes and related structures”. Cilium movement and assembly were suppressed at both levels of analysis, indicating the significant impacts of FHV infection on host ciliary structures. Cilia play a vital role in respiratory defense by clearing pathogens and foreign particles through ciliary movement [40,41]. The dual-level suppression of this function may reduce the ability of the respiratory tract to clear FHV infection, facilitating viral replication and spread. Cytoskeleton-dependent material transport is critical for host cellular metabolism, signaling, and defense. FHV infection may disrupt normal material flow within cells by downregulating these transport pathways, impairing immune signal transduction and further weakening the host’s antiviral capacity. Dynein and axonemal structures are essential for ciliary and cytoskeletal movement [42,43,44,45]. The downregulation caused by FHV infection may directly weaken ciliary motility and cytoskeletal stability, further reducing the host cells’ responsiveness to viral infection. In particular, diminished ciliary motion may lead to reduced pathogen clearance efficiency in the respiratory tract, exacerbating the severity of infection.

For groups treated with AAEO, we summarized the common upregulated terms in transcriptomics and proteomics. Compared to the FHV group, hosts treated with AAEO exhibited significant enhancements in several critical immune-related functions, including the rapid mobilization of immune cells (via the upregulation of chemotaxis and immune cell migration), enhanced coordination between innate and adaptive immunity (upregulation of complement activation, humoral immunity, and MHC class II molecules), and reinforced structural support at infection sites to maintain tissue integrity. These enhanced immune functions suggest that AAEO might enable the host to better resist FHV infection by promptly responding to viral invasion through multilayered immune mechanisms [46,47]. These findings provide clues for anti-infection treatment strategies, particularly by strengthening the complement system, humoral immunity, and immune cell migration and localization to improve host defenses.

In the intersection analysis of downregulated terms, host responses under AAEO treatment exhibited the following characteristics: Inflammation suppression: the dual downregulation of leukocyte and granulocyte migration indicates that the host controlled excessive immune cell aggregation to avoid severe inflammatory responses [48]. Reduced antimicrobial granule release: the downregulation of immune cell granules and lysosomal structures suggests that the host reduced the release of antimicrobial factors to minimize tissue damage caused by the spread of inflammatory mediators [49]. Tissue protection and matrix stability: the downregulation of endopeptidase activity helps reduce extracellular matrix degradation, maintain tissue structural integrity, and prevent self-damage caused by immune responses [50]. These findings highlight AAEO’s role in balancing immune responses to achieve effective pathogen control while protecting host tissues.

In the KEGG pathway analysis, compared to the blank group, the FHV infection group was significantly enriched in the NOD-like receptor signaling pathway and the Toll-like receptor signaling pathway at both transcriptomic and proteomic levels. These two pathways are critical innate immune pathways for recognizing and responding to pathogen invasion [51,52]. The significant enrichment of the NOD-like receptor signaling pathway led to a notable increase in IL-1β, a key pro-inflammatory cytokine. IL-1β is typically upregulated in the IL-17 pathway to enhance inflammation. It further induces more pro-inflammatory factors by activating other inflammatory pathways (e.g., NF-κB) and recruits immune cells to infection sites [53,54]. IL-1β also plays a crucial role in the recruitment and activation of neutrophils [55,56,57]. In the Toll-like receptor signaling pathway induced by FHV infection, a significant increase in IL-1β was similarly observed. This sustained high expression of IL-1β may be a primary cause of FHV-induced inflammation in cats. In addition to IL-1β upregulation, IP-10 (also known as CXCL10) also showed significant upregulation. The upregulation of IP-10 further indicates that, after FHV infection, the host immune system not only triggered an inflammatory response but also activated a strong chemotactic response [58,59]. Studies have also shown that FeHV-1 infection activates the PI3K/Akt/mTOR signaling axis, which contributes to the upregulation of IL-1β and other inflammatory cytokines [60]. This suggests a potential interaction between the PI3K/Akt/mTOR axis and the IL-17 signaling pathway during FeHV-1 infection, amplifying inflammation and tissue damage.

In the comparison between AAEO-treated groups and the FHV infection group, both transcriptomic and proteomic results showed significant downregulation of the IL-17 signaling pathway. The IL-17 signaling pathway plays a vital role in infection and inflammatory responses [61], primarily by promoting the expression of pro-inflammatory cytokines, attracting neutrophils, and enhancing host defense mechanisms to combat pathogens [62,63]. However, excessive IL-17 signaling may lead to persistent inflammation and increase the risk of tissue damage. Therefore, the downregulation of this pathway by AAEO may help alleviate the inflammatory response induced by FHV infection and protect host tissues. Specifically, components of this pathway, including FOSB, MMP1, and IL-1β, showed significant downregulation. FOSB, a member of the Fos family and a component of the AP-1 transcription factor complex, typically forms a complex with Jun proteins to regulate gene expression. FOSB plays a role in various cellular processes (especially inflammatory responses) by activating inflammation-related genes to promote immune responses [64]. MMP1, a member of the matrix metalloproteinase family, degrades the extracellular matrix (ECM) to facilitate immune cell migration and tissue remodeling [65]. In the IL-17 signaling pathway, MMP1 is typically upregulated to help immune cells reach the infection site [66]. Overall, the downregulation of FOSB reduces the expression of pro-inflammatory genes at its source, subsequently inhibiting MMP1 and IL-1β production, thereby minimizing matrix degradation and tissue damage caused by immune cell aggregation. In conclusion, FHV infection activates the NOD-like receptor signaling pathway and Toll-like receptor signaling pathway, significantly upregulating IL-1β and driving inflammatory responses, leading to host tissue damage. Under AAEO treatment, the regulation of the IL-17 pathway suppresses IL-1β expression, thereby controlling the inflammatory response and preventing excessive inflammation-induced damage to the host.

## 6. Conclusions

Our study demonstrates that AAEO is clinically effective in treating FVR and alleviating the inflammatory response caused by FeHV-1 infection in cats. This effect is likely achieved by downregulating IL-1β through the modulation of FOSB in the IL-17 signaling pathway. Additionally, in vitro experiments reveal that AAEO provides protective effects against FHV infection in F81 cells without directly damaging the virus, suggesting that its mechanism of action requires further investigation.

## Figures and Tables

**Table 1:** Top 50 metabolites detected in volatile oil samples of *Artemisia argyi* leaves based on LC-MS and GC-MS dual platforms.

Molecular Formula	Compound	CAS	Molecular Mass (Da)
C_9_H_10_O_3_	Paeonol	552–41–0	166.06
C_10_H_18_O	Endo-Borneol	507–70–0	154.14
C_10_H_18_O	Bicyclo[2.2.1]heptan-2-ol, 1,7,7-trimethyl-, (1S-endo)-	464–45–9	154.14
C_13_H_20_O_3_	Methyl jasmonate	1211–29–6	224.14
C_18_H_30_O_2_	α-Linolenic Acid	463–40–1	278.22
C_10_H_10_O_2_	Safrole	94–59–7	162.07
C_8_H_6_N_2_S	4-Phenyl-1,2,3-thiadiazole	25445–77–6	162.03
C_16_H_32_O	Palmitaldehyde	629–80–1	240.25
C_10_H_22_O	1-Decanol	112–30–1	158.17
C_19_H_36_O_5_	1,2-Dioctanoyl-sn-glycerol	–	344.26
C_10_H_14_O_2_	Nepetalactone trans-cis-form	17257–15–7	166.10
C_14_H_8_O_4_	Danthron; 1,8-Dihydroxyanthraquinone	117–10–2	240.04
C_18_H_30_O_2_	Octadeca-11E,13E,15Z-trienoic acid	25575–00–2	278.22
C_10_H_16_O	(+)-2-Bornanone	464–49–3	152.12
C_10_H_16_O	Camphor	76–22–2	152.12
C_10_H_16_O	5,7-Octadien-4-one, 2,6-dimethyl-, (E)-	6752–80–3	152.12
C_13_H_20_O	Damascenone	23726–91–2	192.15
C_9_H_14_O	Isophorone	78–59–1	138.10
C_8_H_16_O	Octanal	124–13–0	128.12
C_15_H_22_O	Nootkatone	4674–50–4	218.17
C_13_H_20_O	β-Ionone	79–77–6	192.15
C_11_H_22_O_2_	Undecylic Acid	112–37–8	186.16
C_15_H_26_O	Enantio-7(11)eudesmen-4-ol	186374–63–0	222.20
C_15_H_22_O	α-Cyperone	473–08–5	218.17
C_16_H_22_O_4_	Diisobutyl phthalate	84–69–5	278.15
C_15_H_18_O_5_	6α,10α-Dihydroxy-1-oxoeremophila-7(11),8(9)-dien-12,8-olide	–	278.11
C_13_H_20_O	α-Ionone	127–41–3	192.15
C_10_H_18_O	Eucalyptol	470–82–6	154.14
C_16_H_35_NO_2_	2-Aminohexadecane-1,4-diol	–	273.27
C_9_H_18_O	Nonanal	–	142.14
C_15_H_26_O	Elemol	639–99–6	222.20
C_16_H_35_NO_2_	Hexadecylsphingosine	–	273.27
C_12_H_16_O_3_	Elemicin	487–11–6	208.11
C_10_H_18_O_2_	Butanoic acid, 5-hexenyl ester	108058–75–9	170.13
C_10_H_18_O	Terpinen-4-ol	562–74–3	154.14
C_12_H_12_O_3_	Senkyunolide C	–	204.08
C_13_H_24_O	2-Tridecenal	7774–82–5	196.18
C_11_H_16_O_2_	Dihydroactinidiolide	17092–92–1	180.12
C_9_H_12_O_3_	2-(2-Acetoxy-1-propyl)furan, (S)	60830–70–8	168.08
C_10_H_12_O_2_	Eugenol	97–53–0	164.08
C_10_H_18_O	(S)-2,5-Dimethyl-3-vinylhex-4-en-2-ol	35671–15–9	154.14
C_10_H_12_O_2_	Ethyl phenylacetate	101–97–3	164.08
C_11_H_14_O	Isovalerophenone	582–62–7	162.10
C_9_H_10_O	1-Phenyl-1-propanone	93–55–0	134.07
C_10_H_12_O_2_	Isoeugenol	97–54–1	164.08
C_15_H_22_O	Germacrone	6902–91–6	218.17
C_10_H_16_	1,3,6-Octatriene, 3,7-dimethyl-, (Z)-	3338–55–4	136.13
C_10_H_16_	Beta-Phellandrene	555–10–2	136.13
C_10_H_18_O	(+)-Isoborneol	16725–71–6	154.14
C_10_H_22_O	2-Decanol	1120–06–5	158.17

**Table 2:** Clinical scoring system of feline viral rhinotracheitis.

Clinical Signs		Score
Conjunctivitis	None	0
	Mild conjunctival hyperemia	1
	Moderate to severe conjunctival hyperemia	2
	Moderate to severe conjunctival hyperemia and chemosis	3
Blepharospasm	None	0
	Eye < 25% closed	1
	Eye 25–50% closed	3
	Eye completely closed	4
Ocular discharge	None	0
	Minor serous discharge	1
	Moderate mucoid discharge	2
	Marked mucopurulent discharge	3
Body temperature	<39.2 °C	0
	≥39.2 °C	1
Sneezing	None	0
	Observed	1
Nasal discharge	None	0
	Minor serous discharge	1
	Moderate mucoid discharge	2
	Marked mucopurulent discharge	3
Nasal congestion	None	0
	Minor congestion (barely audible)	1
	Moderate congestion (easily audible)	2
	Marked congestion with open-mouth breathing	3
Cough	None	0
	Observed	1
Death		20

**Table 3:** Organizational homogenization program parameter information.

Operation Program	Parameter
Total grinding cycles	2 cycles
Preset grinding frequency	60.5 Hz
Interruption time during grinding	5 s
Grinding run time	120 s

**Table 4:** Automated nucleic acid extraction program.

Process	Mixing Time	Magnetic Attraction Cycles	Mixing Speed	Volume (μL)	Drying Time	Temperature
Transfer magnetic beads	10 s	2	Fast	600	0	Off
Lysis	10 min	5	Fast	800	0	55 °C
Wash 1	30 s	2	Fast	600	0	Off
Wash 2	30 s	2	Fast	600	0	Off
Wash 3	15 s	2	Fast	600	300 s	Off
Elution	10 min	5	Fast	70	0	60 °C
Discard magnetic beads	10 s	0	Fast	600	0	Off

**Table 5:** Real-time PCR primer sequences.

Primer	Sequence (5′-3′)
FHV-qF-DNA-1215	AGAGGCTAACGGACCATCGA
FHV-qR-DNA-1215	GCCCGTGGTGGCTCTAAAC
FHV-qP-1215	TATATGTGTCCACCACCTTCAGGATCTACTGTCGT

## Data Availability

The data supporting the conclusions of this article are included within the article. Additional data used and/or analyzed during the current study are available from the corresponding author upon reasonable request.
